# Hypersensitive K303R oestrogen receptor-α variant not found in invasive carcinomas

**DOI:** 10.1186/bcr965

**Published:** 2004-11-23

**Authors:** Michael PA Davies, Penny A O'Neill, Helen Innes, D Ross Sibson

**Affiliations:** 1 Clatterbridge Cancer Research Trust, JK Douglas Laboratories, Clatterbridge Hospital, Bebington, Wirral, UK; 2 Clatterbridge Centre for Oncology, Clatterbridge Hospital, Bebington, Wirral, UK

**Keywords:** breast, carcinoma, hyperplasia, mutation, oestrogen receptor

## Abstract

**Introduction:**

Genetic abnormalities or mutations in premalignant breast lesions may have a role in progression toward malignancy or influence the behaviour of subsequent disease. The A908G (Lys303→Arg) change in the gene encoding oestrogen receptor-α (ER-α) creates a hypersensitivity to oestradiol and would have significant consequences if present in breast carcinoma, especially those treated with endocrine therapy. We have therefore examined a panel of endocrine-treated invasive carcinomas for the presence of this mutation.

**Methods:**

Sequencing of control DNA was shown to detect mutation present in as little as 15% of the starting material. Enrichment for the mutation by using *Mbo*II restriction digestion allowed the detection of mutant present at 1% or less. We applied these techniques to genomic DNA and cDNA from 136 invasive breast carcinomas.

**Results:**

No evidence of the A908G mutation was found with either technique. The incidence of this mutation in our panel of tumours is therefore significantly less than previously reported.

**Conclusion:**

The fact that the mutation was not found leads us to believe that this mutation is absent from most cells in invasive carcinomas and furthermore that the major expression product of the ER-α gene in cancers does not contain the K303R mutation. It is therefore unlikely to influence the effectiveness of endocrine treatment.

## Introduction

Most breast cancers overexpress oestrogen receptor-α (ER-α), and this molecule is the major target of the anti-estrogens, such as tamoxifen, used to treat the disease. Not all patients respond to anti-oestrogen treatment and many who do respond later relapse. The reasons for treatment failure are still unclear. The suggestion that mutation of ER-α might have a role in the formation of breast cancer and subsequent response to treatment was raised by the detection of a somatic A908G (Lys303→Arg; K303R) mutation in the gene encoding ER-α. This mutation was reported in a significant proportion of breast hyperplasia [1] and also in the majority of invasive cancers and all metastases tested [2,3]. The K303R ER-α variant apparently exhibits a hypersensitivity to oestradiol [1], a characteristic that might allow breast cancers to respond to much lower levels of oestrogenic stimulation with a subsequent impact on malignant progression and the effectiveness of anti-oestrogen treatment. This would be of particular importance in post-menopausal women and in women receiving anti-oestrogen therapy. We have therefore studied a cohort of post-menopausal, endocrine-treated breast cancers for the presence of this mutation. Furthermore, because expression of the hypersensitive mutant would be required for activity, we also examined mRNA.

The detection of mutations in breast cancers is not a trivial task, owing in part to the heterogeneity of tumour tissue and also to the reporting of false positive and false negative results. Although microdissection allows the purity of selected cellular populations to be enhanced, the sensitivity and specificity of standard DNA sequencing approaches still limit our ability to assess mutation status accurately. We now show that by performing a second round of sequencing after enrichment for the mutation, we were able to increase the sensitivity and specificity of our assay and apply it to invasive carcinomas without the need for microdissection.

## Methods

### Determination of sensitivity for mutation detection

To confirm the sensitivity of this technique for detection of the mutant allele in the presence of wild-type DNA, experiments were performed with the use of mixtures of cloned wild-type and mutant polymerase chain reaction (PCR) products, generated with primers described by Fuqua and colleagues [1] (ERmut1, 5'-CAA GCG CCA GAG AGA TGA TG-3'; ERmut2, 5'-ACA AGG CAC TGA CCA TCT GG-3').

Plasmid clones of PCR products with either an A or a G at position 908 of ER-α were mixed in various ratios (100% to 0% mutant) and diluted to the equivalent of 1000 copies per PCR reaction before being amplified and sequenced. A two-stage PCR was performed on 2 μl of mixed DNA in the 20 μl first-round reaction (20 cycles) and 4 μl of first-round product in the 40 μl second round (50 cycles). For each PCR an initial 13 min 95°C denaturation step was followed by cycles of 95°C for 30 s, 68°C for 60 s and a single 3 min final extension at 72°C. Other PCR conditions were also the same in both rounds: 0.2 mM dNTPs, each primer at 0.5 μM, 0.5 units of HotstarTaq DNA polymerase (Qiagen) and 1× PCR Buffer (containing 1.5 mM MgCl_2_; Qiagen).

**Figure 1:**
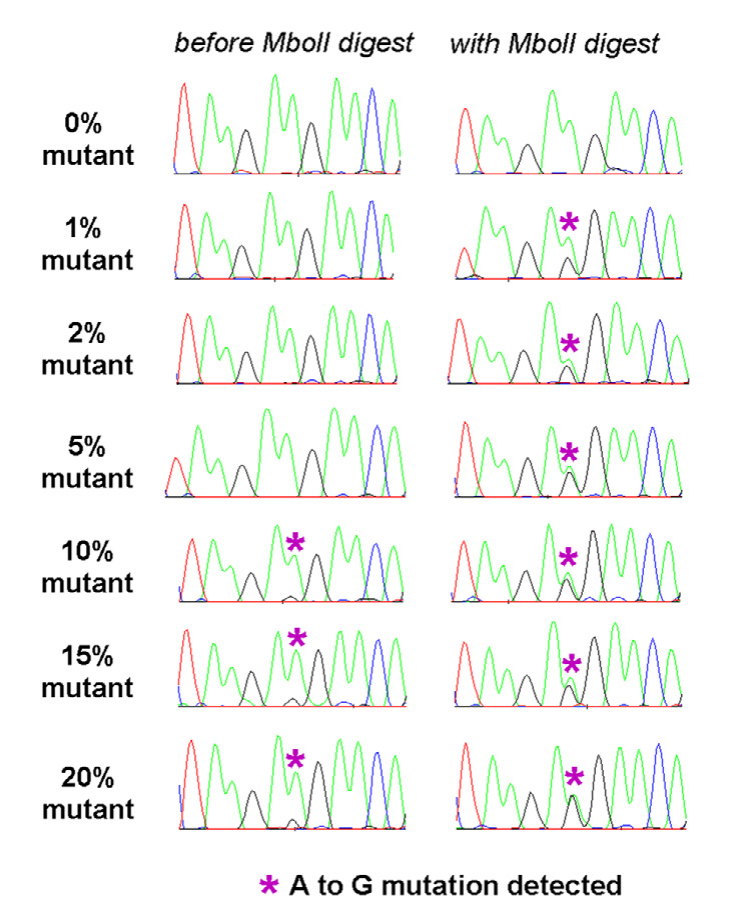
Sample electrophoretograms of sequence data with and without enrichment by digestion with *Mbo*II. The sequence of the region of interest is shown: coding residues 903–913 (residues 1195–1205 of *ESR1*; GenBank accession number M12674). The position of the detected A908G mutation is marked with asterisks.

PCR products were sequenced (Fig. 1) by using primers ERmut1 and ERmut2 and subjected to *Mbo*II digestion, reamplification and further sequencing as described below.

### Digestion with *Mbo*II and reamplification by PCR

**Figure 2:**
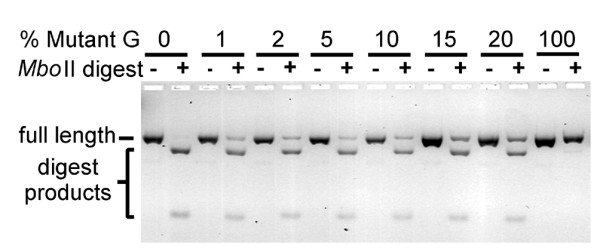
Agarose gel electrophoresis of control PCR products with and without digestion with *Mbo*II.

The *Mbo*II restriction enzyme has a GAAGA recognition site; this sequence is present at the K303R mutation with the second A being mutated to G. Digestion with *Mbo*II can therefore be used as an assay for the presence of mutation, with non-digested DNA indicating mutation within the recognition site [4]. PCR product (5–10 μl) was digested in a total volume of 20 μl including 1× Buffer2 (New England Biolabs) and 5 units of *Mbo*II restriction enzyme (New England Biolabs) by incubation at 37°C for 90 min. PCR products were separated by electrophoresis on gels containing 3% Seakem Agarose (Flowgen) and TAE buffer (40 mM Tris-acetate, 1 mM EDTA, pH 7.6). Molecular mass markers (φX174/*Hae*III; Abgene) were included on each gel and DNA was revealed by the inclusion of 0.5 μg/ml ethidium bromide, scanning with a Molecular Dynamics FluorimagerSI and analysis with ImageQuant version 4.1 software (Molecular Dynamics). After digestion the non-mutant 158-base-pair (bp) ERmut1–ERmut2 PCR product gives rise to 118-bp and 40-bp bands, detectable by agarose gel electrophoresis (Fig. 2), whereas 158-bp PCR products containing mutant remain undigested. Similarly ERADNA1–ERADNA2 genomic DNA and ERARNA1–ERARNA2 cDNA PCR products give different patterns of digestion products depending on the presence of a mutation affecting the *Mbo*II recognition site.

Rather than relying on the detection of non-digested PCR products by agarose gel electrophoresis, we further increased the sensitivity and specificity of this assay by sequencing amplified non-digested DNA after digestion. PCR reamplification of non-digested DNA (20 cycles) was performed as above for primers ERmut1 and ERmut2 with 2 μl of a 1:10 dilution of the restriction digest. Reamplified PCR products were sequenced with ERmut1 primer. In all cases with any evidence of a mutant G at the relevant position, alternative PCR reactions were examined and reverse sequencing with primer ERmut2 was performed, to rule out PCR or sequencing anomalies.

### ER-α PCR

For *ESR1 *genomic DNA (RefSeq NM_000125) from breast cancer cases, primers were designed that were specific for genomic DNA (because they included intronic sequence) and that better allowed sequence determination in both directions (ERADNA1, 5'-AAG TGG CCT GAA GTT TGT TA-3'; ERADNA2, 5'-CTT ACC TGG CAC CCT CTT-3'). PCR reactions contained 1 ng of genomic DNA, 0.2 mM dNTPs, each primer at 1 μM, 0.5 units of HotstarTaq DNA polymerase (Qiagen), 1× PCR Buffer (containing 1.5 mM MgCl_2_; Qiagen) and additional MgCl_2 _to a final concentration of 2.5 mM. An initial 13 min 95°C denaturation step was followed by 40 cycles of 94°C for 40 s, 60°C for 40 s, 72°C for 60 s and a single 10 min final extension at 72°C. The 609-bp genomic PCR product is digested by *Mbo*II to give a 296-bp product, a 6-bp product and products of 130 and 177 bp; the last two of these fail to digest if a mutation is present and instead result in a 307-bp product that can be reamplified by ERmut1 and ERmut2.

For RNA analysis from breast cancer cases, reverse transcription (RT) was performed in duplicate on 0.5 μg of RNA in accordance with the manufacturer's instructions (Invitrogen), after a DNAaseI digestion step (Invitrogen). RT reactions incorporated Superscript II Reverse Transcriptase (Invitrogen), 0.5 μg of oligo (dT)_17 _and 0.5 μl of Prime Recombinant Ribonuclease Inhibitor (Eppendorf). Parallel reactions were performed in which the RT enzyme was omitted; these acted as controls for genomic DNA contamination. RT–PCR reactions were performed with cDNA-specific primers designed to cross intron–exon boundaries (ERARNA1, 5'-AAG TGG GAA TGA TGA AAG GT-3'; ERARNA2, 5'-CAA GAG CAA GTT AGG AGC AA-3') and that better allowed sequence determination in both directions. These primers are located in exons 4 and 6 of ER-α and generate a 491-bp RT–PCR product. The same primers occasionally amplify additional, minor, shorter products arising from splice variants in which exon 5 is absent. PCR reactions contained 2 μl of a 1:20 dilution of the RT reaction, 0.2 mM dNTPs, each primer at 1 μM, 0.5 units of HotstarTaq DNA polymerase (Qiagen) and 1× PCR Buffer (containing 1.5 mM MgCl_2_; Qiagen). PCR cycling conditions were the same as for genomic DNA from breast cancers. The 491-bp genomic PCR product is digested by *Mbo*II to give a 135-bp product, a 49-bp product and products of 130 bp and 177 bp; the last two of these fail to digest if a mutation is present and instead result in a 307-bp product that can be reamplified by ERmut1 and ERmut2.

### DNA sequencing

PCR products were treated with ExoSAP (Amersham Biosciences) before sequencing. Fluorescent DNA sequencing (Fig. 1) was performed with DYEnamic ET Dye Terminator Cycle Sequencing Kit for MegaBACE (Amersham Biosciences) and analysed on a MegaBACE 1000 (Amersham Biosciences). For RT–PCR products, the primers used for PCR were also used for sequencing. When sequencing the genomic DNA from breast cancers the reverse primer ERADNA2 and an additional forward sequencing primer (ERADNA3, 5'-TAC GAA AAG ACC GAA GAG-3') were used. Primer ERADNA3 is located in exon 5 of ER-α and overcomes difficulties in sequencing through an A and T rich tract in intron 4 of ER-α; the same primer can also be used to sequence RT–PCR products and overcomes any difficulties caused by exon-5-deleted PCR products. DNA sequencing of genomic and cDNA PCR products was performed in both directions and the position of the putative mutation was analysed individually for each PCR product. To call a mutation we adopted the standard practice that it should be observed in at least two independent PCR reactions (avoiding possible PCR misincorporation due to *Taq *polymerase infidelity) and in both sequencing directions (avoiding sequencing anomalies due to misincorporation of dideoxy terminators).

### Patients and samples

Post-menopausal patients undergoing treatment for invasive breast cancer during the period between 1993 and 1999 were identified within the database of the Cancer Tissue Bank Research Centre (CTBRC) at the Royal Liverpool University Hospital. The diagnoses of invasive cancers were made in accordance with the pathology guidelines of the NHS Breast Screening Program [5].

DNA and/or RNA from 136 cases were selected for analysis and provided by CTBRC along with further details as given in Table 1. All tissue donated to CTBRC is fully consented for research purposes and permission was granted by all required local research ethics committees.

## Results

Our ability to detect variable amounts of mutant 908G ER-α in the presence of wild-type 908A ER-α was confirmed by sequencing PCR products from mixtures of these two variants (Fig. 1). Direct sequencing was routinely sensitive enough to detect mutant ER-α when present in as little as 15% of the starting DNA and was frequently able to detect the mutation at even lower levels (for example 10%). This was true of sequencing in either direction, although for the original primers described by Fuqua and colleagues [1] the reverse sequence was more difficult to read because the mutation was closer to the reverse PCR primer (ERmut2). The new primers designed for use on cDNA overcame this limitation, while ensuring that only cDNA was amplified. PCR primers for genomic DNA were similarly specific for genomic DNA and, when used in conjunction with a sequencing primer (ERADNA3), again gave sequence in both directions.

Digestion with *Mbo*II restriction enzyme allowed the detection of as little as 1% mutant DNA by agarose gel electrophoresis in control reactions (Fig. 2). However, no evidence of undigested PCR product was visible for any breast tumour assayed in this way. When sequencing control DNA reamplified with ERmut1 and ERmut2 after enrichment for mutant DNA by *Mbo*II digestion (Fig. 1), the mutant G base was clearly detected even when only present in 1% of the original DNA. The wild-type A was also detected, either because of inefficient digestion or because of heterodimer formation in the PCR reaction. Notably in control reactions containing only wild-type ER-α, any post-digest reamplified DNA was clearly wild type. Therefore, despite a failure to detect non-digested PCR product on gels stained with ethidium bromide, reamplification and sequencing confirms that restriction digestion is seldom 100% efficient and that by PCR we are able to reamplify products originating from 1% or less of the starting DNA. It is therefore possible to apply this reamplification and sequencing technique to all samples, because the PCR used routinely amplifies non-digested DNA enriched for mutant PCR product.

The enhanced sensitivity after enrichment using digestion with *Mbo*II and reamplification is not without drawbacks, in that we detected a greater number of PCR and sequencing anomalies with this technique. For non-enriched sequence analysis, evidence of a very minor G in genomic DNA from two breast cancers was subsequently shown to be due to sequencing anomalies because they were not present either in repeat PCR products from the same cases or in sequence generated in the reverse direction. After enrichment, similar errors were noted in eight genomic DNA PCR products and six cDNA PCR products. Ten of these anomalies were apparently due to *Taq *polymerase infidelity (that is, they were identified in reverse sequencing but not repeatable in replicate PCR) and four were sequencing anomalies (that is, they were not identifiable in reverse sequencing). Notably, errors due to *Taq *polymerase infidelity were seen only with the more sensitive assay based on enrichment with *Mbo*II, and no such anomalies of either type were seen in any non-mutant controls at the relevant base position.

We were unable to confirm that the proposed ER-α A→G mutation leading to a K303R amino acid substitution was present in any case of invasive cancer examined. In all, we examined 136 cases of invasive cancer (Table 1) with our *Mbo*II-enriched sequencing assay, having also sequenced 130 of these before enrichment. With the exception of the 14 cases noted previously, in every sequenced PCR product the A at the base position of the variant was clearly an A with no evidence of any G substitution. For 60 of these cases we examined both genomic DNA and cDNA, for 71 cases genomic DNA only was examined (for example, for ER-α-negative cancers) and for 5 cases only cDNA was examined. All 100 cases of invasive cancer for which a pathologist assessed the proportion of tumour cells contained at least 50% tumour, and 63% contained at least 90% tumour cells. Given the proven sensitivity of our techniques we would therefore have expected to detect the hypersensitive ER-α mutation even if present in only a minority of cancer cells or contaminating normal cells.

## Discussion

The abundant expression of ER-α in most breast cancers is fundamental to both our understanding of this disease and its treatment. The observations that ER-α is overexpressed in a proportion of premalignant lesions [6,7] and is possibly related to an increased risk of progression [8] further raise the importance of oestrogenic activity in the establishment and behaviour of breast carcinoma. It is therefore important to understand whether other mechanisms for increased ER-α activity are also present in breast tumours or their putative precursors. One such mechanism is the hypersensitivity apparently inferred by a K303R mutant ER-α reported to be present in a significant proportion of breast hyperplasia [1].

We have been unable to detect the reported A908G mutation of ER-α in genomic DNA from any case of invasive carcinoma in our study, despite applying a sensitive and robust assay capable of detecting mutant if present in as little as 1% of the starting DNA. The absence of this hypersensitive ER-α variant suggests that this mutation is not present in the majority of cells in such lesions, or indeed even in a significant minority. Furthermore, the absence of the mutation in RT–PCR products confirms that non-mutant ER-α is the major expression product in the breast cancers. Zhang and colleagues also failed to detect the mutation in a variety of breast lesions from Japanese women [4], and while this manuscript was under review two further papers have reported a lack of evidence for the K303R mutation [9,10]. We have previously been unable to detect the mutation by sequencing a series of 56 microdissected ductal carcinomas *in situ *from an unrelated cohort, confirming that this mutation is also apparently absent from these premalignant lesions.

Case selection can influence detection rates, and the cohort of patients tested here was selected by virtue of being post-menopausal and treated with endocrine therapies but not chemotherapy, so as to allow any effect on endocrine treatment to be more easily defined. Although it is possible that the mutation occurs in other groups of breast cancers, our cohort is broadly representative of subgroups defined by ER-α status, nodal status, grade, tumour size and histology. Other studies reporting an inability to detect the mutation [4,9,10] have applied no obvious selection criteria and it therefore seems unlikely that any selection bias is to blame for the lack of detectable mutation.

The low level of risk of subsequent cancer attached to hyperplasia implies that many of these lesions fail to contribute to disease progression and it is perhaps therefore unsurprising that a mutation so far reported in only about one-third of hyperplasias by a single laboratory is found to be absent from more advanced lesions in the present study. Preliminary reports of the K303R mutation in a higher proportion of breast cancers [2,3] from the same group as reported the original observations in hyperplasia are perplexing. Although confirmatory data have not so far been published, these results continue to be reported at scientific meetings and referenced in reviews [11]. Many previous studies of mutation in breast cancers have failed to report this mutation, and more recent reports [4,9,10] have also produced evidence that if it exists it does so below the level of detection of the techniques used. It is therefore important to consider the sensitivity and specificity of the different assays used to detect the K303R mutant. The fact that Zhang and colleagues analysed 7 breast hyperplasias [4] and Tebbit and colleagues analysed 25 hyperplasias [9] and found no mutation rules out the use of hyperplasia as 'positive' controls and challenges the original observations.

Here the sequencing of PCR products with fluorescent dideoxy terminators is shown to be sensitive enough to detect a 908G mutant when present at 15% or less of the starting DNA, in keeping with the results of Tebbit and colleagues [9]. This is probably suitable for use with microdissected lesions as used by Tebbit and colleagues for 36 invasive cancers, or lesions with a relatively high tumour content as used here (Table 1), but might not be sensitive enough for use with non-microdissected cancers used elsewhere [4,10] or if the mutation is present in only a small minority of cancer cells. We therefore also used gel-based PCR with restriction-fragment-length polymorphism and were able to detect as little as 1% mutant by agarose gel electrophoresis of PCR products from control mixing experiments. Although the same technique was used by Zhang and colleagues for 215 cancers [4], they did not report their detection sensitivity or confirm their specificity. Given that this technique potentially detects very low levels of mutation at any position in the *Mbo*II recognition site, it is important to confirm sensitivity and specificity by a further round of DNA sequencing. A very significant enhancement of sequencing sensitivity (to 1% mutant or less) was seen with this *Mbo*II digestion and reamplification technique. We can therefore state that this mutation is not present in the vast majority of tumour cells of any cancer tested, even without the benefit of enrichment for tumour cells by microdissection.

Although assay sensitivity is important, specificity is equally crucial. We never reported any mutant in either assay used for our non-mutant control DNA, indicating that our specificity is high. However, in our experience, false positive mutations by DNA sequencing, as seen here in 16 of the 136 cancers tested, are to be expected when PCR reactions are performed on very low levels of DNA as found in many microdissected samples, or after digestion with restriction enzyme. It is therefore important to perform rigorous confirmatory assays. To this end, we always sought to confirm mutations by sequencing in both directions to avoid anomalies that often occur as a result of the misincorporation of dideoxy terminators in unidirectional sequencing. We also sequenced multiple PCR products to avoid anomalies due to *Taq *polymerase infidelity. Originally, the K303R mutation was reported in 34% of microdissected hyperplasias tested by unidirectional, non-fluorescent sequencing [1]. This assay is prone to false positives and it is unclear whether replicate PCR or other confirmatory assays were performed. The fact that the mutation was always found in addition to the wild type is also to be expected with artefacts due to dideoxy terminator misincorporation. The possibility that observations of the K303R mutation in hyperplasia result from a lack of specificity must therefore be considered and some doubt cast over other positive results in cancers.

## Conclusion

We have developed and validated a highly sensitive and specific assay for the detection of the A908G (K303R) mutation of ER-α. Having tested 136 different breast tumours, we find no evidence to support the hypothesis that the A908G mutation of ER-α is present or expressed in breast carcinoma cells from post-menopausal, endocrine-treated patients. It is therefore unlikely to have an impact on the effectiveness of such treatments.

## Abbreviations

ER-α = oestrogen receptor-α; PCR = polymerase chain reaction; RT = reverse transcription.

## Competing interests

The author(s) declare that they have no competing interests.

## Authors' contributions

All authors listed contributed to the production of this manuscript. PAO and HI provided clinical review of cases studied; PAO prepared the cDNA used; HI provided genomic DNA; mutation detection was performed by MPAD, and DRS provided both technical insight and critical review. The manuscript was produced by MPAD. All authors read and approved the final manuscript.

## Figures and Tables

**Table 1:** Case selection

Parameter	Detail	Value
Age (years)	Range	48–88
Histology	Invasive ductal	115
	Invasive lobular	11
	Other	10
ER-α	Positive	88
	Negative	42
Grade	1	21
	2	53
	3	62
Nodal status	Positive	51
	Negative	62
Size	<2 cm	55
	≥ 2 cm	79
Stage	1	33
	2	76
	3	5
Tumour (%)	≥ 50	100
	≥ 75	91
	≥ 90	63

ER-α, oestrogen receptor-α.
